# Impact of device variability and protocol differences on kidney function during normothermic machine perfusion: A comparative study using porcine and human kidneys

**DOI:** 10.1111/aor.14851

**Published:** 2024-08-28

**Authors:** Veerle A. Lantinga, Asel S. Arykbaeva, Nora A. Spraakman, Elwin W. P. Blom, Tobias M. Huijink, Dorottya K. de Vries, Rutger J. Ploeg, Ian P. J. Alwayn, Henri G. D. Leuvenink, Cyril Moers, L. Leonie van Leeuwen

**Affiliations:** ^1^ Department of Surgery, University Medical Center Groningen University of Groningen Groningen the Netherlands; ^2^ Department of Surgery Leiden University Medical Center Leiden the Netherlands; ^3^ Department of Anaesthesiology, University Medical Center Groningen University of Groningen Groningen the Netherlands; ^4^ Nuffield Department of Surgical Sciences University of Oxford Oxford UK; ^5^ Recanati/Miller Transplantation Institute Icahn School of Medicine at Mount Sinai New York City New York USA

**Keywords:** kidney transplantation, normothermic machine perfusion, perfusion device

## Abstract

**Introduction:**

A growing interest in renal normothermic machine perfusion (NMP) has resulted in more clinically available perfusion devices. While all perfusion systems have the same aim, there are significant differences in their circuits, pumps, sensors, and software. Therefore, our objective was to assess the impact of different perfusion protocols and devices on kidney function and perfusion parameters during NMP.

**Methods:**

Porcine kidneys were subjected to 30 min of warm ischemia, 24 h of static cold storage, and subsequently perfused for 6 h using (1) the Kidney Assist (KA) machine with a pressure of 75 mm Hg, (2) the KA device incorporating several adjustments and a pressure of 85 mm Hg (modified KA), or (3) the Perlife (PL) perfusion device (*n* = 4). Consecutively, discarded human kidneys were perfused using the KA or modified KA (*n* = 3) protocol.

**Results:**

The PL group quickly reached the device’s upper flow limit and consequently received a significantly lower pressure compared to the KA groups. The arterial pO_2_ was significantly lower in the PL group. Yet, hemoglobin concentration increased over time, and oxygen consumption was significantly higher compared to the KA groups. Fractional sodium excretion was significantly lower in the PL group. Tissue ATP levels, urine production, and creatinine clearance rates did not differ between groups. In human kidneys, the modified KA group showed significantly lower vascular resistance, higher oxygen delivery, and lower levels of lactate in the perfusate compared to the KA group.

**Conclusions:**

This study shows that perfusion characteristics and kidney function are significantly influenced by the perfusion protocol and the device and its settings during normothermic machine perfusion and therefore should be interpreted with caution.

## Graphical abstract

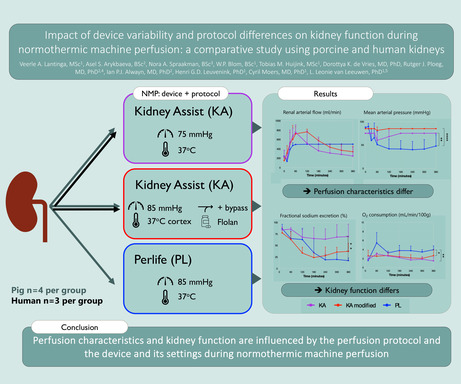

## 1 INTRODUCTION

The worldwide shortage of donor kidneys has led to an increase in the acceptance of suboptimal grafts. Outcomes after a kidney transplant obtained from extended criteria donors are less favorable than grafts from standard criteria donors, with an increased incidence of delayed graft function and graft loss within six months.^1^ Objective evaluation of kidney grafts before transplantation is still challenging, as the available donor characteristics at the time of organ offer poorly predicted transplant outcomes.^2^

During ex vivo normothermic machine perfusion (NMP), the kidney is perfused at 37°C with a red blood cell‐based perfusate containing oxygen and nutrients, mimicking in vivo physiology. Compared to standard, low temperature (<10°C) kidney graft preservation,^3^, ^4^ rewarming the kidney to normothermic temperatures (35–37°C) restores metabolism and might allow functional evaluation of the graft.^5^, ^6^, ^7^ Studies concerning liver and lung NMP have already suggested the value of this technique in assessing organ viability by successfully increasing the number of liver and lung transplantations.^8^, ^9^, ^10^ Due to its potential, renal NMP is rapidly gaining interest.

Several clinical trials were initiated to investigate the safety and feasibility of renal NMP.^5^, ^6^, ^11^, ^12^, ^13^ Viability markers predicting transplant outcomes have been proposed; however, these have not yet been validated.^6^ As the physiology of a donor kidney during NMP is not well understood, the optimal conditions for the organ, such as perfusate composition, perfusion pressure, and oxygen delivery, are still under debate.^14^, ^15^ There is no consensus about the best protocol for renal NMP, and currently centers use different protocols.^14^, ^15^

Not only protocols, but also perfusion devices used for NMP differ per center. Until recently, the Kidney Assist (KA) (XVIVO, Göteborg, Sweden) was the only commercially available and CE‐marked normothermic perfusion device for donor kidneys. Other reported set‐ups are custom‐made systems using cardiopulmonary bypass devices for extracorporeal circulation.^6^ A new commercially available CE‐marked device entered the market two years ago: the PerLife (PL) system, designed by Aferetica, San Giovanni in Persiceto, Italy. While all machines have similar aims, there are significant differences in the perfusion circuit, measurement techniques, and software of these devices. As all devices are either pressure‐ or flow‐controlled, accurate flow and pressure measurements are essential for NMP. Also, the use of different arterial cannulas (e.g., based on the presence or absence of an aortic patch) could result in significant deviations in measurements, which impacts the resulting pressure in the renal artery and downstream vasculature. Nevertheless, the extent to which different circuits, measurement techniques, and cannulas influence perfusion characteristics and kidney function during NMP has never been described.

During our first attempts to perform NMP with discarded human donor kidneys using the Kidney Assist, subphysiological perfusion flows and temperatures were observed. This led to hypoperfusion and, consequently, suboptimal temperature control and low oxygen delivery to the tissue, potentially damaging the organ. These observations indicated that our protocol required improvement.

Our aim was to assess the impact of different perfusion protocols and devices on perfusion parameters and kidney function during NMP. We first evaluated the effect of three different NMP protocols on perfusion parameters and kidney function during NMP of porcine kidneys. Hereby comparing two different perfusion devices. We then tested the perfusion protocol that we found to be superior on human discarded donor kidneys, aiming to improve the perfusion characteristics that we had observed in our previous experiments with human kidneys.

## 2 MATERIALS AND METHODS

### 2.1 Experimental design

**FIGURE 1:**
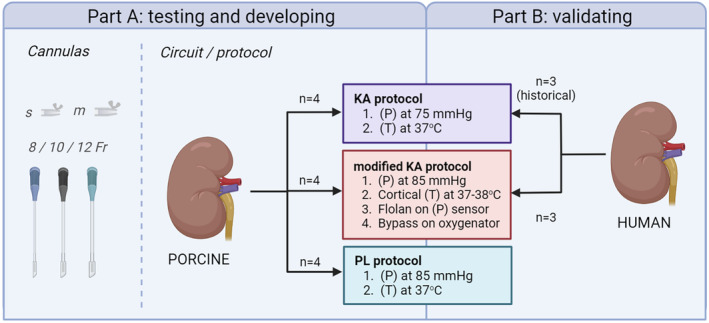
Experimental design. Fr, French; KA, Kidney Assist; KA+WA, Kidney Assist with workaround; (P), pressure setting; PL, perlife; m, medium; s, small; (T), temperature setting. [Color figure can be viewed at wileyonlinelibrary.com]

To investigate the impact of a particular perfusion device, we evaluated two distinct perfusion devices: the Kidney Assist and the PerLife system. Additionally, to test the effect of different perfusion protocols, we refined our current NMP protocol using the Kidney Assist, using a higher‐pressure setting, changing the location of the infusion of a vasodilator, improving temperature monitoring, and altering the perfusion circuit by adding a shunt line. In part A, these three perfusion setups were tested using porcine kidneys (Figure 1). Furthermore, we tested the effect of using different perfusion cannulas. In part B, the modified KA protocol was tested using discarded human kidneys and compared to our historical cohort consisting of discarded human kidneys that were previously perfused.

### 2.2 Part A: Developing and comparing NMP protocols using porcine kidneys

#### 2.2.1 Cannulation

A commonly used cannula for renal NMP is a metal patch holder (HAGMED, Warsaw, Poland), available in sizes small, medium, and large. Alternatively, a straight cannula (HAGMED, Warsaw, Poland) can be used for kidneys with a calcified or absent aortic patch, available in the sizes 8/10/12 Fr. We compared these cannulas and calculated the pressure drop caused by each cannula with respect to different places of pressure measurements in the circuit. The details are described in the supplementary methods.

#### 2.2.2 Perfusion experiments

##### 2.2.2.1 Organ procurement and preservation

Twelve viable kidneys were retrieved *post‐mortem* from six‐month‐old Dutch Landrace pigs at a local abattoir, following all guidelines of the Dutch food safety authority as previously described.^16^, ^17^, ^18^ These kidneys had an average weight of 244 ± 17 g. All kidneys were exposed to 30 min of warm ischemia followed by an immediate, gravitational arterial flush (±80 mm Hg) with 500 mL ice‐cold University of Wisconsin cold storage solution (UW‐CS; Belzer MPS, Bridge to Life, London, UK). Afterward, kidneys were stored on ice in 500 mL UW‐CS for 24 h.

##### 2.2.2.2 NMP protocols

**FIGURE 2:**
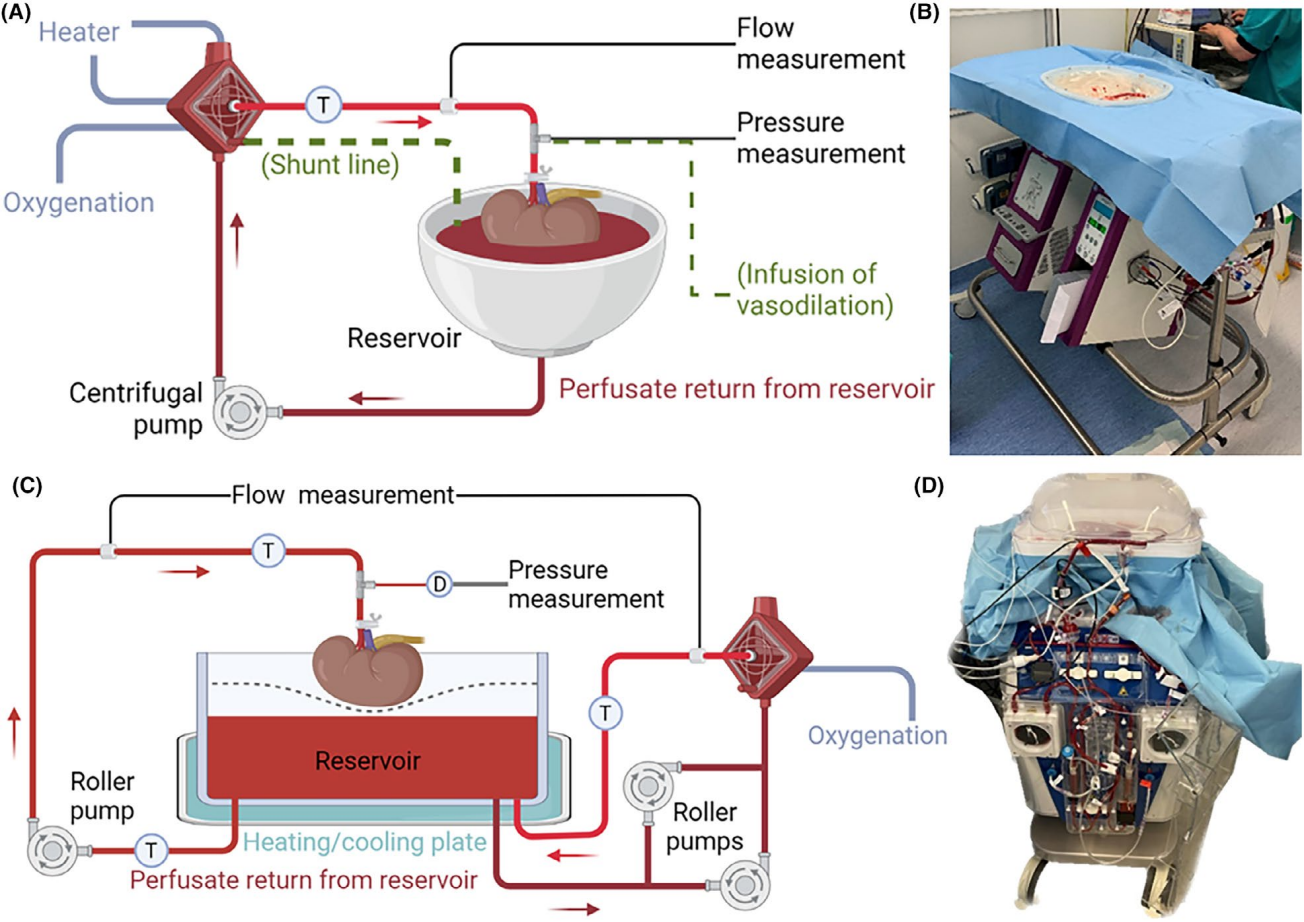
Perfusion setup. (A). Schematic overview of Kidney Assist perfusion setup. The extra shunt line and the infusion of vasodilation of the modified protocol are shown in green. (B). Picture of Kidney Assist perfusion setup. (C). Schematic overview of PerLife perfusion setup. (D). Picture of PerLife perfusion setup. D, pressure dome; T, temperature sensor. [Color figure can be viewed at wileyonlinelibrary.com]

After 24 h of static cold preservation, porcine kidneys were flushed with 50 mL ice‐cold UW‐CS, and the renal artery was cannulated for NMP using a metal patch holder, size S. NMP was performed using either the Kidney Assist (XVIVO, Göteborg, Sweden) or the PerLife system (Aferetica, San Giovanni in Persiceto BO, Italy). The perfusion setup is shown in Figure 2, and the detailed protocol for each group is described in the supplementary methods. The composition of the perfusate and the infusions used during NMP are shown in Table S1. All setups were oxygenated using carbogen (95% oxygen, 5% CO_2_) at a flow rate of 500 mL/min. Urine, perfusate, and biopsies were sampled at various time points during NMP. Urine was recirculated to the perfusate after sampling.^19^ Renal cortex temperature was monitored using an infrared thermometer (SP95, STREX, Deventer, The Netherlands). Renal flow rate, mean arterial pressure (MAP), renal resistance, perfusate temperature, renal cortex temperature, and urine production were continuously recorded during NMP.

The perfusion circuits for the modified KA and PL groups were fitted with an extra clinical‐grade disposable TruWave pressure transducer (Edwards Lifesciences, Irvine, USA) as close to the kidney as possible and a calibrated ultrasonic flow sensor (Transonic, Ithaca, USA) clamped on corresponding tubing to allow measurement of renal arterial flow and mean arterial pressure in an equal manner between groups.

### 2.3 Part B: Comparing protocols using discarded human kidneys

Six human deceased donor kidneys, deemed unsuitable for transplantation, were obtained after refusal by all transplant centers within the Eurotransplant region, and after consent for research had been provided by the donors' next of kin. Human kidneys had an average weight of 171 ± 37 g. Donor characteristics and reasons for discard are shown in Table S2.

Three discarded human kidneys (obtained between January and March 2021) were perfused as extensively described before by Arykbaeva et al.^14^ (NCT04693325) and are referred to as the Kidney Assist human kidneys historical cohort. The other three human discarded kidneys (obtained between July and August 2022) were perfused according to our modified Kidney Assist protocol, as described above, and are referred to as KA modified.

### 2.4 Renal function and viability assessment for part A and B

Creatinine and sodium concentrations in urine and perfusate samples, lactate dehydrogenase (LDH), aspartate aminotransferase (ASAT), and the hemolysis index in perfusate samples, and protein levels in urine samples were analyzed in a standardized manner by a clinical chemistry laboratory (UMCG, Groningen, The Netherlands). Additionally, partial oxygen pressure, hemoglobin content, saturation, and pH were measured using an ABL90 FLEX PLUS blood gas analyzer (Radiometer, Zoetermeer, The Netherlands). Equations for calculating oxygen delivery, oxygen consumption, metabolic coupling, and fractional sodium excretion are shown in Table S3.

For adenosine triphosphate (ATP) analysis, cortical biopsies were homogenized in ice‐cold sonication solution (70% ethanol and 2 mM EDTA). Supernatants were analyzed using an ATP Bioluminescence Kit CLS II (Roche Diagnostics, Mannheim, Germany). ATP values were normalized to protein content measured using a Pierce™ BCA Protein Assay Kit (Thermo Scientific, Waltham, USA).

Cortical biopsies were fixed in 4% formalin, after which they were dehydrated by immersing tissues in a series of ethanol solutions of increasing concentrations. These tissues were then cleared in xylene, embedded in paraffin wax, and cut into sections of 4 μm. Sections were stained using a Periodic‐acid Schiff (PAS) staining to visualize morphological features. Sections were examined in a blinded fashion, by a highly experienced renal pathologist.

### 2.5 Statistical analysis for part A and B

GraphPad Prism (version 9.1.0) was used to visualize and analyze the data. All data are expressed as the arithmetic mean with the standard deviation (SD) in a longitudinal fashion. Differences across porcine experimental groups were assessed using a one‐way ANOVA followed by a Tukey's multiple comparison test, and for human experimental groups using an unpaired Student's *t*‐test. Differences between sensors were assessed using a paired Student's *t*‐test. All statistical tests were two‐tailed, and differences between groups were considered statistically significant when *p* < 0.05.

## 3 RESULTS

### 3.1 Part A: Developing and comparing NMP protocols using porcine kidneys

#### 3.1.1 Pressure loss during NMP is influenced by the cannula used

**FIGURE 3:**
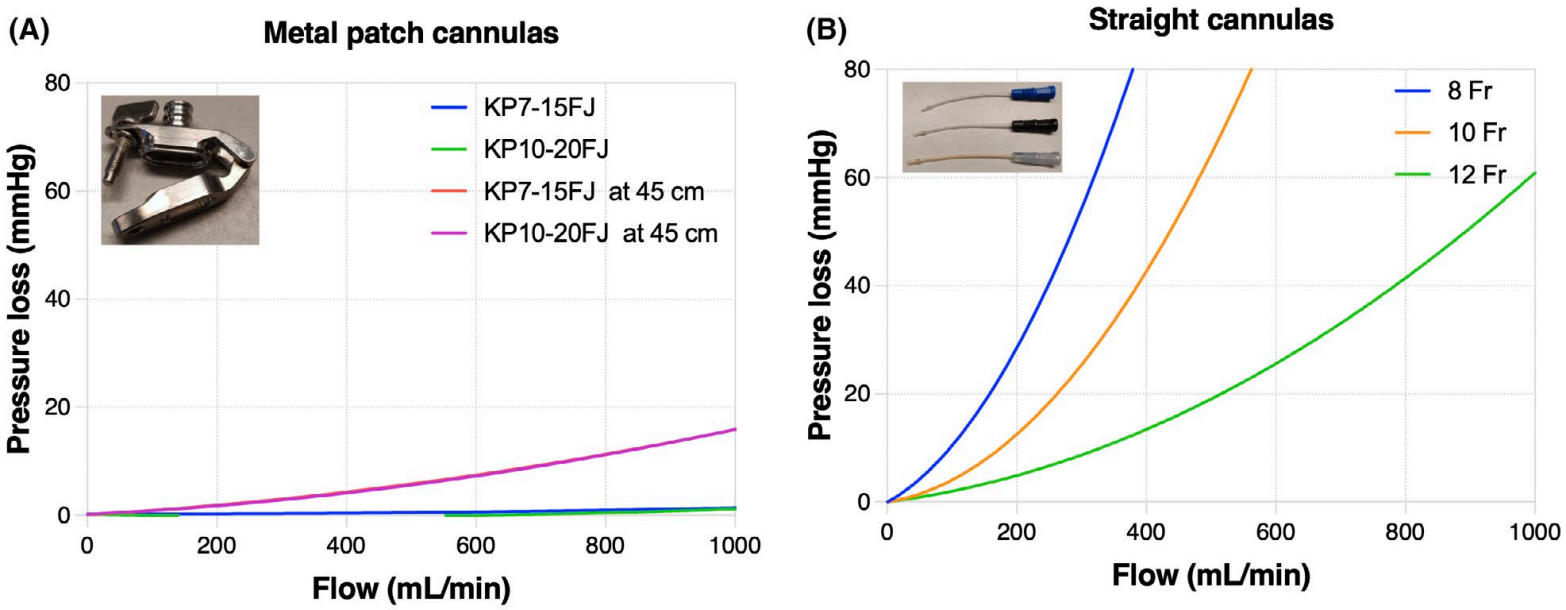
Pressure drop versus flow rate for different cannulas. Measured for the (A) small (KP7‐15FJ) and medium (KP10‐20FJ) metal patch cannula recommended when using the Kidney Assist or PerLife perfusion device, and (B) 8, 10, and 12 French straight cannulas recommended by the manufacturers when lacking an arterial patch. [Color figure can be viewed at wileyonlinelibrary.com]

First, pressure drop versus flow rate was tested for the cannulas recommended for both clinical devices used. Pressure loss caused by metal patch cannulas proved to be minimal (Figure 3A). When the pressure sensor was placed 45 cm from the cannula (similar to the Kidney Assist circuit), pressure loss was higher compared to a setup where the pressure was measured closer to the cannula. In contrast, a considerable pressure loss, resulting in a much lower actual perfusion pressure of the kidney, occurred when straight cannulas were used (Figure 3B).

#### 3.1.2 Different NMP protocols influence perfusion parameters

**FIGURE 4:**
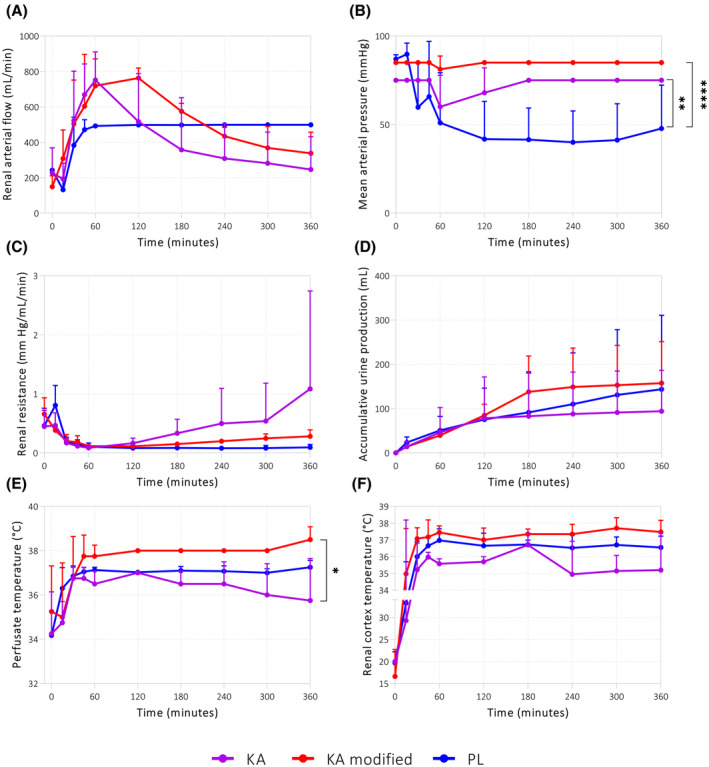
Perfusion parameters during NMP. As shown by (A) the renal arterial flow, (B) mean arterial pressure, (C) the renal resistance, (D) the accumulative urine production, (E) perfusate temperature, and (F) the renal cortex temperature. *p < 0.05, **p < 0.01, and ****p < 0.0001. Values are expressed as mean ± SD (*n* = 4 kidneys per group). KA, Kidney Assist; PL, PerLife. [Color figure can be viewed at wileyonlinelibrary.com]

Next, perfusion parameters per protocol were compared. The flow of the kidneys in the PL group plateaued after 1 h of NMP when all perfused kidneys had reached the 500 mL/min upper flow limit (Figure 4A), resulting in a MAP of 40–50 mm Hg (Figure 4B). The flow within the KA and modified KA groups showed a different trend, increasing during the first 2 h of perfusion and slowly decreasing afterward (Figure 4A). The 1000 mL/min flow limit was reached for several kidneys in the KA and the modified KA groups after 1 h of NMP, resulting in an inevitable perfusion pressure drop (Figure 4B). The MAP in the PL group was significantly lower compared to the KA groups. The significant differences in MAPs did not result in significant differences in renal resistance (Figure 4C). The accumulative urine production did not differ between groups (Figure 4D). Perfusate temperature was significantly higher in the modified KA group than in the KA group, but the renal cortical temperature was similar for all groups (Figure 4E,F).

#### 3.1.3 The location of the oxygenator influences oxygenation

**FIGURE 5:**
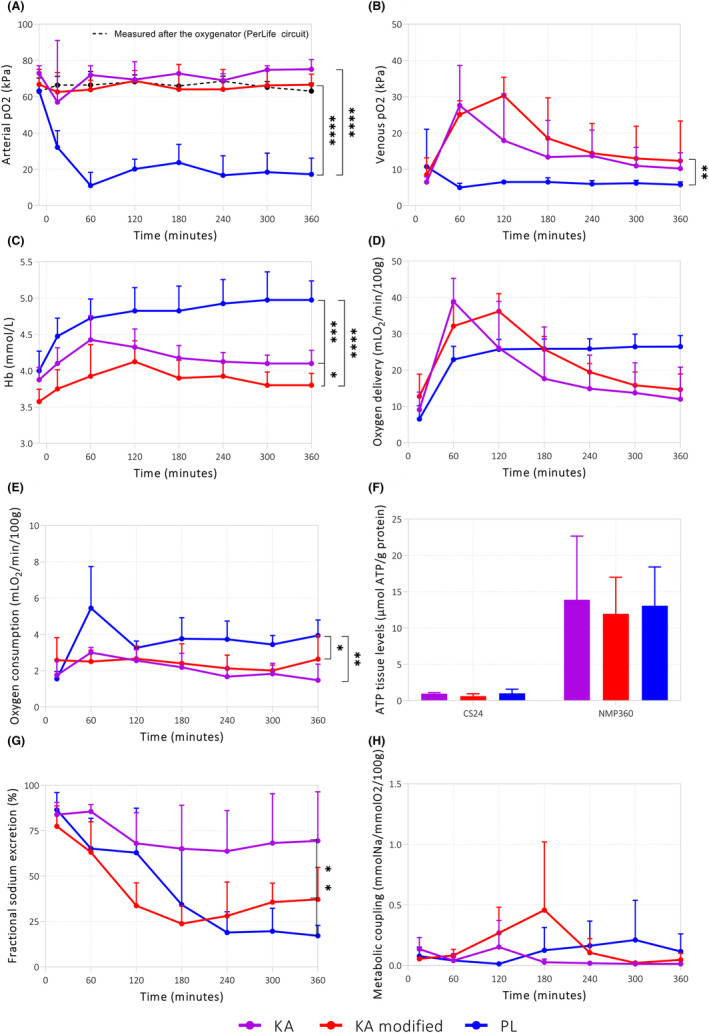
Oxygenation and metabolism during NMP. As measured by the (A) arterial partial oxygen pressure, (B) venous partial oxygen pressure, (C) arterial hemoglobin levels, (D) oxygen delivery, (E) oxygen consumption, and (F) renal cortex ATP levels, (G) fractional sodium excretion, and (H) metabolic coupling. Values were measured at the arterial sample port. *p < 0.05, **p < 0.01, ***p < 0.001, and ****p < 0.0001. Values are expressed as mean ± SD (*n* = 4 kidneys). KA, Kidney Assist; PL, PerLife. [Color figure can be viewed at wileyonlinelibrary.com]

Another remarkable difference between the perfusion setups is the location of the oxygenator. In the PL setup, the oxygenator is in a parallel circuit, while for the KA, the oxygenator is positioned in series (Figure 1A,B) with and upstream to arterial perfusate flow. This difference resulted in a significantly lower arterial and venous pO_2_ in the PL group (Figure 5A,B). Interestingly, the mean Hb in the PL group was significantly higher compared to that in the KA groups—albeit this is presumably not a result of oxygenator location (Figure 5C). Much as there were no significant differences in oxygen delivery, oxygen consumption was significantly higher in the PL group compared to the KA groups (Figure 5D,E). No significant differences in tissue ATP were observed between groups pre‐ and post‐NMP (Figure 5F). Looking at renal function, the fractional sodium excretion was significantly lower in the PL and modified KA groups compared to the KA group (Figure 5G). The significant differences in oxygen consumption and fractional sodium excretion did not lead to significant differences in metabolic coupling (Figure 5H).

#### 3.1.4 Different NMP protocols do not lead to different injury profiles

**FIGURE 6:**
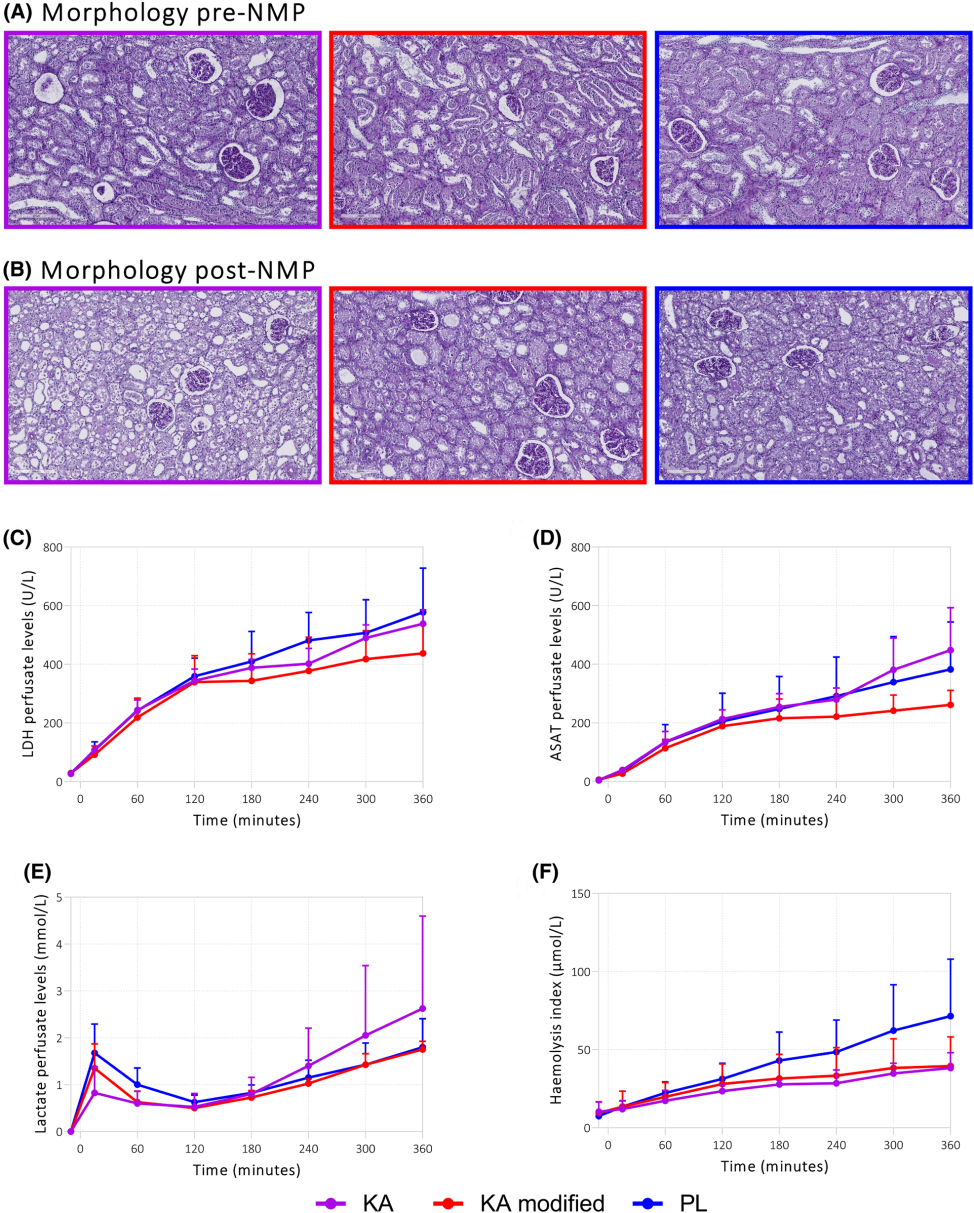
Renal morphology and injury during NMP. As shown by the (A) general tissue morphology pre‐NMP, and (B) post‐nmp (200× magnification) (C) LDH levels in the perfusate, (D) ASAT levels in the perfusate, (E) lactate levels in the perfusate, (F) the hemolysis index. Values are expressed as mean ± SD (*n* = 4 kidneys). KA, Kidney Assist; PL, PerLife. [Color figure can be viewed at wileyonlinelibrary.com]

Blinded histological assessment of renal cortical tissue revealed no morphological differences between experimental groups or between pre‐ and post‐NMP when globally looking at glomerular dilation and tubular dilation and necrosis (Figure 6A,B). No significant differences in general injury markers were observed (Figure 6C–E). Hemolysis levels also did not differ per group (Figure 6F).

#### 3.1.5 Flow and pressure values differ when using different sensors and perfusion devices

**FIGURE 7:**
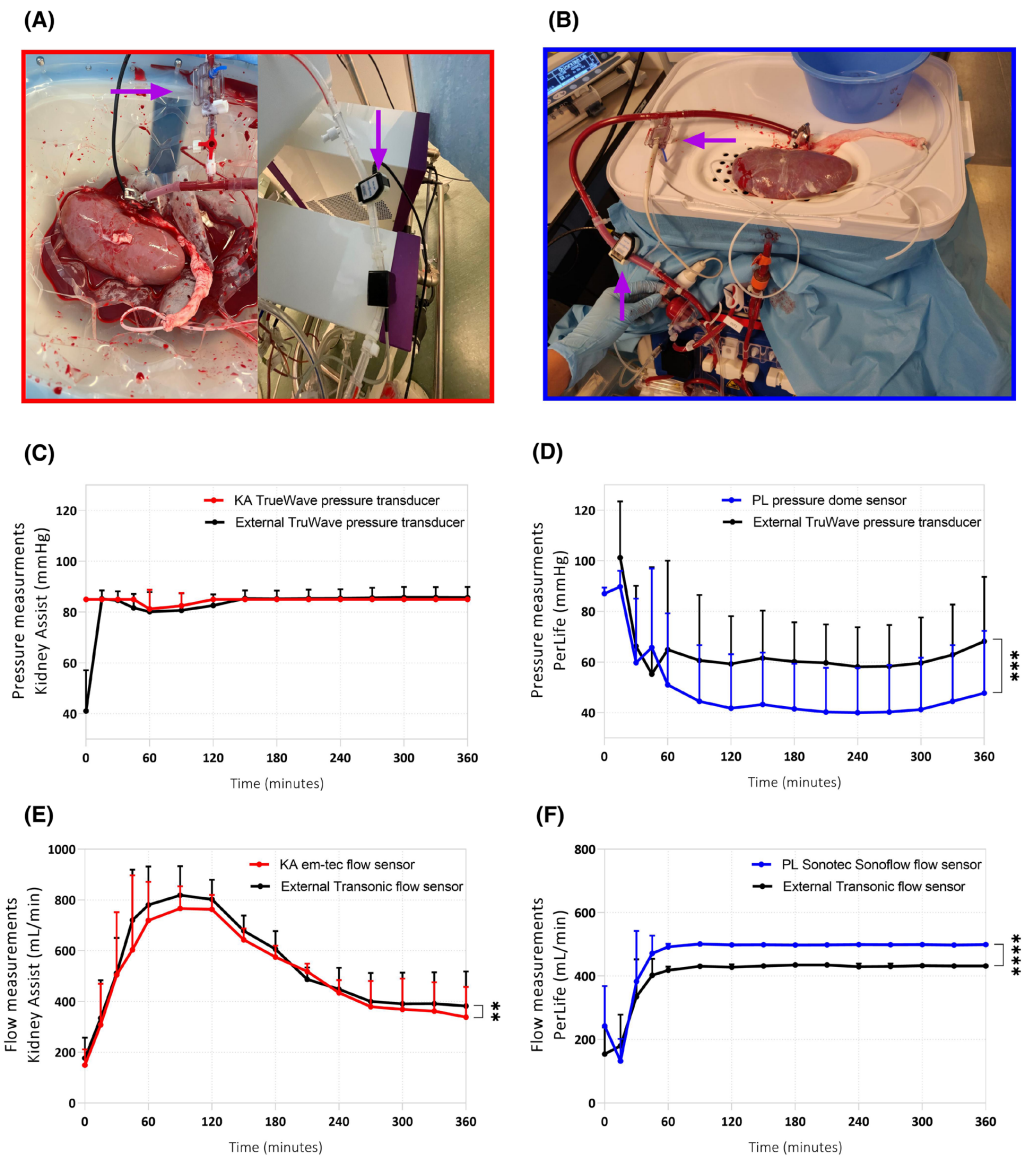
External pressure and flow measurements. Measured by an external TruWave pressure transducer and an external Transonic flow sensor added to the (A) Kidney Assist circuit and (B) PerLife circuit. (C) Pressures measured in the Kidney Assist circuit, (D) pressures measured in the PerLife circuit, (E) flow measured in the Kidney Assist circuit, and (F) flow measured in the PerLife device circuit. **p < 0.01, ***p < 0.001, and ****p < 0.0001. Values are expressed as mean ± SD (*n* = 4 kidneys per group). KA, Kidney Assist; PL, PerLife. [Color figure can be viewed at wileyonlinelibrary.com]

Since the KA and the PL measure flow and pressure using different techniques, each machine was fitted with an additional, very accurate, and calibrated flow and pressure sensor to compare these techniques. The PL system uses an air‐filled pressure dome connected to the inlet tube approximately 50 cm from the renal artery. The KA system uses a fluid‐primed pressure line without a pressure dome at about 15 cm from the renal artery. A second pressure sensor was connected close to the renal artery to validate these measurements in both systems (Figure 7A,B). There was no significant difference in pressure measurement between the original setup of the KA and the extra pressure sensor (Figure 7C). The pressure measured in the original setup of the PL was significantly lower than the pressure measured with the extra pressure sensor (Figure 7D), with a difference of ±20 mm Hg. An external flow sensor was added to validate flow measurements in each set‐up (Figure 7A,B). The external transonic flow sensor measured a significantly different flow compared to the traditional sensors of the KA and the PL (Figure 7E,F).

### 3.2 Part B: Comparing protocols using discarded human kidneys

#### 3.2.1 The modified protocol significantly improves both flow and temperature

**FIGURE 8:**
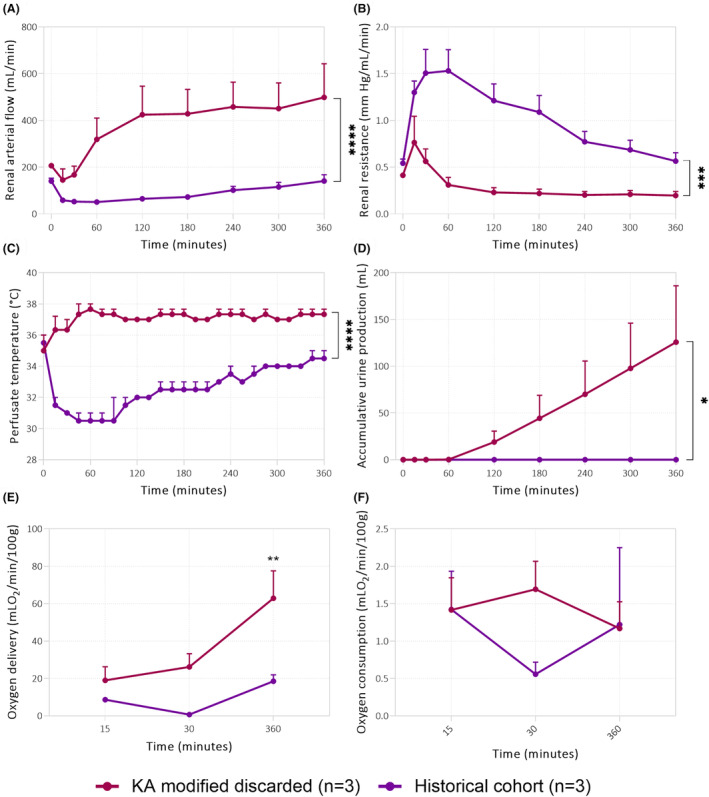
Perfusion parameters during pre‐clinical NMP. As shown by (A) the renal arterial flow, (B) renal resistance, (C) perfusate temperature, (D) accumulative urine production, (E) oxygen delivery, and (F) oxygen consumption. *p < 0.05, **p < 0.01, ***p < 0.001, and ****p < 0.0001. Values are expressed as mean ± SD (*n* = 3 kidneys). HC, historical cohort; KA, Kidney Assist; WA, workaround. [Color figure can be viewed at wileyonlinelibrary.com]

As the modified KA protocol seemed to help sustain a desired flow and temperature during NMP, we then tested this protocol using discarded human kidneys. Perfusate flow of kidneys perfused with the modified KA protocol was significantly higher, and mean renal resistance was significantly lower compared to the historical cohort (Figure 8A,B). The temperature of the perfusate in the reservoir was significantly lower in the KA group compared to the modified KA group (Figure 8C). Urine production was significantly higher in the modified KA group, as there was no urine output in the historical cohort (Figure 8D). Oxygen delivery only significantly differed at 6 h of perfusion (Figure 8E). No differences were observed in oxygen consumption (Figure 8F).

#### 3.2.2 The modified protocol results in lower arterial lactate levels

**FIGURE 9:**
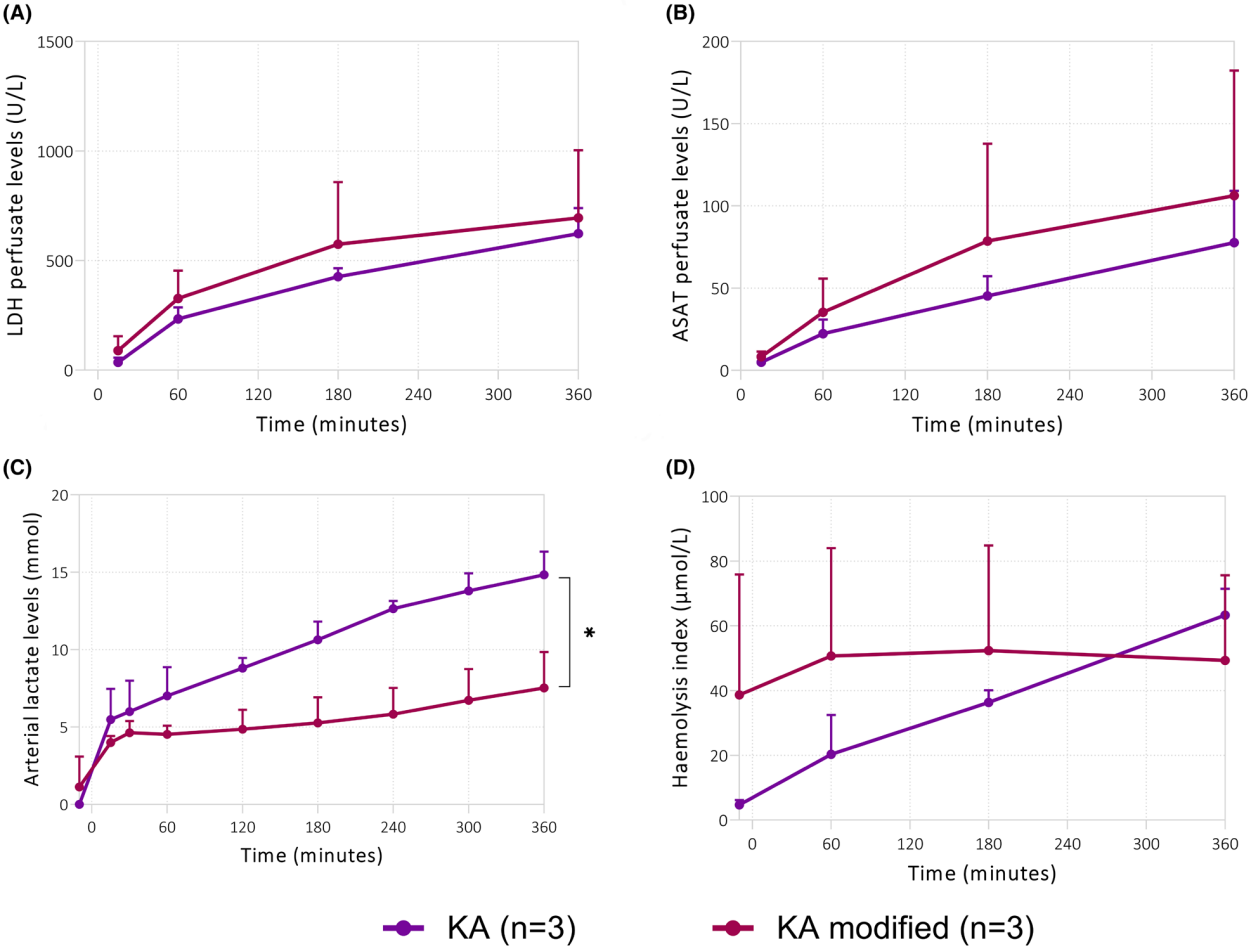
Injury markers during pre‐clinical NMP. As shown by the (A) LDH perfusate levels, (B) ASAT perfusate levels. (C) Arterial lactate levels, (D) hemolysis index. *p < 0.05. Values are expressed as mean ± SD (*n* = 3 kidneys). KA, Kidney Assist; WA, workaround. [Color figure can be viewed at wileyonlinelibrary.com]

Last, but not least, we analyzed injury markers. We found no differences in perfusate LDH and ASAT levels between groups (Figure 9A,B). Kidneys perfused with the modified KA protocol had significantly lower arterial lactate levels (Figure 9C). No differences in hemolysis were observed (Figure 9D).

## 4 DISCUSSION

To our knowledge, we are the first to meticulously evaluate the PerLife device for renal NMP, compare two clinically approved NMP devices, and demonstrate that both the protocol and the device used significantly affect measured parameters during renal NMP. Our results show that the type of perfusion device and the protocol used have a considerable impact on the course of NMP, influencing both perfusion characteristics and kidney function. For young, healthy pig kidneys with inherent low vascular resistance, the modified protocol only optimized perfusion temperature and had no additional effect on perfusate flow or injury markers. However, for discarded human donor kidneys with higher vascular resistance, the modified protocol significantly improved flow, temperature control, and arterial lactate levels. These findings suggest that standardization of protocols and devices is essential for ensuring consistent outcomes and optimizing the success of NMP.

We first analyzed the effect of different cannulas available for renal NMP. Different cannulas will result in different degrees of friction and turbulence of the perfusion fluid, leading to a varying resistance to flow.^20^, ^21^ The longer and thinner the cannula, the higher the pressure drop and the lower the resulting real pressure in the renal artery will be.^22^ This is a well‐known physiological phenomenon, and within cardiothoracic perfusion flow limitations for each cannula are considered. Although different cannula sizes matching the size of the renal artery are recommended by the manufacturers, little information is provided about correcting the pressure setting of the device to ensure an adequate pressure in the renal artery. All reported renal NMP set‐ups are pressure‐controlled, resulting in a perfusion flow that is dependent on the pressure setting. However, we found that simply setting the pressure on each of the two NMP devices investigated does not result in the desired pressure in the renal artery if the lumen of the cannula is small or long. Especially for grafts without an aortic patch using a straight cannula, pressure loss due to the cannula is a major issue. Renal vascular resistance changes over time, and with this the pressure drops. Pressure corrections per cannula are not incorporated in the software, and continuous adjustment of the setting would be necessary to achieve stable perfusion. Also, correcting for a pressure loss up to 60–80 mm Hg at higher flows is not possible with all devices due to a software‐based limitation of this setting. Despite the impact on the pressure in the artery, the technical aspects of the cannulas used in renal NMP studies are barely mentioned. Only in liver perfusion, a group from Zurich described the same phenomenon and designed their own cannula, aiming to minimize pressure loss.^23^ Our results highlight that, if possible, it should be avoided to use long cannulas with a small lumen during NMP in a clinical setting.

Not only the cannula used, but also the perfusion device itself influences renal flow and pressure. One remarkable finding in our study was that the software‐embedded upper flow limit of both devices could considerably alter the course of a perfusion. After reaching the upper flow limit of 500 mL/min using the PL device, perfusion continued with a significantly lower pressure compared to the groups perfused with the KA device. The upper flow limit of 1000 mL/min on the KA machine was reached only occasionally during the first three hours of perfusion, thereby also lowering the perfusion pressure. We observed a dynamic trend in flow through kidneys perfused with the KA, while that of the PL remained stable at the maximum plateau of 500 mL/min throughout perfusion. This remarkable difference in flow trend could be a result of the difference in device operation or differences caused by homeostatic autoregulation of the renal blood flow as a response to the different pressures kidneys were exposed to.^24^ In a physiological setting, the kidneys receive roughly 20%–25% of cardiac output to ensure adequate filtration of blood. However, the desired flow range during NMP has not yet been defined.

Another major difference between the devices in this study is the location of the oxygenator in the circuit. This resulted in a significantly lower arterial pO_2_ value in the PL group compared to the KA group. Despite the lower arterial pO_2_, oxygen delivery was similar due to the higher Hb levels in the PL group. Surprisingly, oxygen consumption was higher in the PL group. This could be explained by the fact that kidneys exposed to higher hemoglobin levels can extract and use a higher proportion of the oxygen that is delivered to the tissue.^25^ Fractional sodium excretion was significantly lower in the PL group compared to the KA group, suggesting that the high oxygen consumption in the PL group is linked to increased tubular function.^26^ Quite contrary to what was expected, a higher oxygen consumption did not lead to differences in metabolic coupling or ATP levels.

Our study found that beside the device, also the protocol will influence perfusion characteristics and kidney function. For example, looking at the renal temperature, the KA software is programmed to prevent overshoot in temperature and slows down heating rate as the temperature approaches its target value (37°C in most protocols). However, the perfusate will cool down after the connection of a cold kidney. Hypothermic conditions cause vasoconstriction, increasing vascular resistance and hampering rewarming of the organ. We hypothesized that this vicious circle could be prevented by temporarily increasing the device's target temperature to 39°C just after the connection of the kidney. Our modified protocol improved temperature control, stabilizing perfusion temperature at 37–38°C. As the rate of metabolism is strongly correlated with temperature, this might influence a kidney's functionality during NMP.^27^

For porcine kidneys with low vascular resistance, there was no significant difference in perfusate flow between the KA and the modified KA protocol. However, for discarded human donor kidneys, vascular resistance of kidneys perfused with the modified protocol was significantly lower, resulting in a higher perfusate flow and, consequently, increased oxygen delivery. The arterial lactate levels were significantly lower in the modified KA protocol compared to the KA protocol for human discarded kidneys. As lactate is a well‐known marker for ischemia, it could be speculated that this higher flow contributed to improved tissue oxygenation. This higher perfusate flow could be due to vasodilation caused by the improved temperature control, leading to vasodilation. However, the infusion site of the vasodilating agent may also have played a role. In the protocol used for the historical cohort, this vasodilator was administered through the sample port (Figure 2), rather distant from the renal artery, while in kidneys perfused with the modified protocol, the vasodilating agent was administered through the pressure line, much closer to the renal artery. Given the very short (3–6 min) half‐life of epoprostenol, these kidneys may have had better exposure to the active vasodilating component of this drug. As a decrease in vascular resistance has been linked to better early graft function, these findings suggest that the modified protocol may be beneficial for improving renal preservation during NMP.^28^

The present study had several limitations. The sample size is small, limiting the generalizability and increasing the risk of statistical errors. We expect little variation between the pigs; however, this does not apply to the discarded human kidneys. The discarded human donor kidneys of the historical cohort in Part B did not produce urine during NMP. Consequently, we could not compare the effect of the modified protocol on functional markers such as fractional sodium excretion between the modified KA group and the historical cohort. Furthermore, it is important to note that the kidneys were not transplanted in this study. Even when functional markers were available, it remains unknown to what extent they predict transplant outcomes and kidney viability. Also, besides the potential advantage of a higher flow and a higher oxygen delivery, there are also potential disadvantages to our modified protocol. Introducing a new shunt in the KA circuit could increase hemolysis, as the inevitably required higher pump rotation speed may damage red blood cells in the perfusate.^29^ However, no difference in hemolysis was observed between kidneys perfused with the KA device with and without the modified protocol for both porcine and human kidney experiments. Only kidneys perfused with the Perlife system showed a notably higher level of hemolysis. Literature shows that the pump type (roller vs. centrifugal) does not necessarily influence hemolysis levels.^30^ However, the higher hemolysis levels in the PL group could be explained by the use of a second and third roller pump in the oxygenation circuit, exposing red blood cells to much more cumulative mechanical forces than a single centrifugal pump (in the KA) will do. Despite the higher hemolysis index, Hb levels were higher in the PL group. The use of a separate oxygenation circuit may have caused pooling and sedimentation of RBCs in the original circuit, resulting in erroneously measured higher hematocrits and Hb levels since perfusate samples were drawn from this original circuit.

The implementation of renal NMP is rapidly rising, and at the time of writing, several clinical trials are ongoing (NCT05031052, NCT04693325, and NCT04882254). The first clinical trials and preclinical research show acceptable results, and tentative suggestions have been made for viability markers during NMP.^7^ These include perfusate flow and urine production. However, our results show that the device and protocol used will strongly influence perfusion characteristics and kidney function on the pump. This suggests that caution should be taken with implementing these viability markers using a different protocol. To ensure consistent outcomes and improve the success of renal transplantation following NMP, future research should focus on optimizing and standardizing protocols and devices.

Renal NMP has been a topic of research as a promising method for preserving and improving the quality of donor kidneys before transplantation. However, it is scarcely implemented clinically as the “perfect” protocol has yet to be described.^15^ We conclude that the use of different perfusion devices and protocols can considerably influence observations during renal NMP in terms of perfusion characteristics and kidney function markers. Results from studies using different protocols or devices should be compared with caution. We show that by modifying the perfusion protocol, a significantly higher flow, better temperature control, and lower arterial lactate levels could be obtained compared to a historical NMP protocol. However, a clinical trial is necessary to analyze the real impact of this modified protocol and the use of different devices on transplant outcomes.

## AUTHOR CONTRIBUTIONS

VAL, ASA, NAS, EWPB, TMH and LLvL performed the experiments and data analysis. VAL and LLvL wrote the manuscript. ASA, NAS, EPWB, TMH, DKdV, RJP, IPJA, HGDL, and CM critically revised the manuscript.

## FUNDING INFORMATION

Although perfusion disposables and machine support were provided to us free of charge, Aferetica and XVIVO had no influence on study design, experiments, data analysis, and reporting of this study's results.

## CONFLICT OF INTEREST STATEMENT

The authors have nothing to disclose.

## Supporting information


**Data S1.**.

## References

[1] Ojo AO . Expanded criteria donors: process and outcomes. Semin Dial. 2005;18(6):463–468. 10.1111/j.1525-139X.2005.00090.x 16398707

[2] Riley S , Zhang Q , Tse W‐Y , Connor A , Wei Y . Using information available at the time of donor offer to predict kidney transplant survival outcomes: a systematic review of prediction models. Transpl Int. 2022;35:10397. 10.3389/ti.2022.10397 35812156 PMC9259750

[3] Fo B , Jh S . Principles of solid‐organ preservation by cold storage. Transplantation. 1988;45(4):673–676. 10.1097/00007890-198804000-00001 3282347

[4] Brat A , de Vries KM , van Heurn EWE , Huurman VAL , de Jongh W , Leuvenink HGD , et al. Hypothermic machine perfusion as a national standard preservation method for deceased donor kidneys. Transplantation. 2022;106(5):1043–1050. 10.1097/TP.0000000000003845 34172648 PMC9038234

[5] Hosgood SA , Nicholson ML . First in man renal transplantation after ex vivo normothermic perfusion. Transplantation. 2011;92(7):735–738. 10.1097/TP.0b013e31822d4e04 21841540

[6] Hosgood SA , Saeb‐Parsy K , Hamed MO , Nicholson ML . Successful transplantation of human kidneys deemed untransplantable but resuscitated by ex vivo normothermic machine perfusion. Am J Transplant. 2016;16(11):3282–3285. 10.1111/ajt.13906 27273794 PMC5096065

[7] Hosgood SA , Thompson E , Moore T , Wilson CH , Nicholson ML . Normothermic machine perfusion for the assessment and transplantation of declined human kidneys from donation after circulatory death donors. Br J Surg. 2018;105(4):388–394. 10.1002/bjs.10733 29210064 PMC5887977

[8] van Leeuwen OB , de Vries Y , Fujiyoshi M , Nijsten MWN , Ubbink R , Pelgrim GJ , et al. Transplantation of high‐risk donor livers after ex situ resuscitation and assessment using combined hypo‐ and normothermic machine perfusion: a prospective clinical trial. Ann Surg. 2019;270(5):906–914. 10.1097/SLA.0000000000003540 31633615

[9] van Leeuwen OB , Bodewes SB , Lantinga VA , Haring MPD , Thorne AM , Brüggenwirth IMA , et al. Sequential hypothermic and normothermic machine perfusion enables safe transplantation of high‐risk donor livers. Am J Transplant. 2022;22(6):1658–1670. 10.1111/ajt.17022 35286759 PMC9325426

[10] Ghaidan H , Fakhro M , Andreasson J , Pierre L , Ingemansson R , Lindstedt S . Ten year follow‐up of lung transplantations using initially rejected donor lungs after reconditioning using ex vivo lung perfusion. J Cardiothorac Surg. 2019;14(1):125. 10.1186/s13019-019-0948-1 31262311 PMC6604441

[11] Minor T , von Horn C , Gallinat A , Kaths M , Kribben A , Treckmann J , et al. First‐in‐man controlled rewarming and normothermic perfusion with cell‐free solution of a kidney prior to transplantation. Am J Transplant. 2020;20(4):1192–1195. 10.1111/ajt.15647 31599063

[12] Rijkse E , Bouari S , Kimenai HJ , de Jonge J , de Bruin RW , Slagter JS , et al. Additional normothermic machine perfusion versus hypothermic machine perfusion in suboptimal donor kidney transplantation: protocol of a randomized, controlled, open‐label trial. Int J Surg Protoc. 2021;25(1):227–237. 10.29337/ijsp.165 34708171 PMC8499718

[13] Hosgood SA , Callaghan CJ , Wilson CH , Smith L , Mullings J , Mehew J , et al. Normothermic machine perfusion versus static cold storage in donation after circulatory death kidney transplantation: a randomized controlled trial. Nat Med. 2023;29(6):1511–1519. 10.1038/s41591-023-02376-7 37231075 PMC10287561

[14] Arykbaeva AS , de Vries DK , Doppenberg JB , Engelse MA , Hankemeier T , Harms AC , et al. Metabolic needs of the kidney graft undergoing normothermic machine perfusion. Kidney Int. 2021;100(2):301–310. 10.1016/j.kint.2021.04.001 33857572

[15] Hamelink TL , Ogurlu B , De Beule J , Lantinga VA , Pool MB , Venema LH , et al. Renal normothermic machine perfusion: the road toward clinical implementation of a promising pretransplant organ assessment tool. Transplantation. 2022;106(2):268–279. 10.1097/TP.0000000000003817 33979315

[16] van Leeuwen LL , Venema LH , Heilig R , Leuvenink HGD , Kessler BM . Doxycycline alters the porcine renal proteome and degradome during hypothermic machine perfusion. Curr Issues Mol Biol. 2022;44(2):559–577. 10.3390/CIMB44020039 35723325 PMC8928973

[17] Schutter R , Lantinga VA , Hamelink TL , Pool MBF , Varsseveld OC , Potze JH , et al. Magnetic resonance imaging assessment of renal flow distribution patterns during ex vivo normothermic machine perfusion in porcine and human kidneys. Transpl Int. 2021;34(9):1643–1655. 10.1111/tri.13991 34448269 PMC9290094

[18] Venema LH , van Leeuwen LL , Posma RA , van Goor H , Ploeg RJ , Hannaert P , et al. Impact of red blood cells on function and metabolism of porcine deceased donor kidneys during normothermic machine perfusion. Transplantation. 2022;106(6):1170–1179. 10.1097/TP.0000000000003940 34456268 PMC9128616

[19] Weissenbacher A , Lo Faro L , Boubriak O , Soares MF , Roberts IS , Hunter JP , et al. Twenty‐four‐hour normothermic perfusion of discarded human kidneys with urine recirculation. Am J Transplant. 2019;19(1):178–192. 10.1111/AJT.14932 29758129 PMC6491986

[20] Minakawa M , Fukuda I , Yamazaki J , Fukui K , Yanaoka H , Inamura T . Effect of cannula shape on aortic wall and flow turbulence: hydrodynamic study during extracorporeal circulation in mock thoracic aorta. Artif Organs. 2007;31(12):880–886. 10.1111/j.1525-1594.2007.00481.x 17924991

[21] Lemétayer J , Broman LM , Prahl WL . Flow dynamics and mixing in extracorporeal support: a study of the return cannula. Front Bioeng Biotechnol. 2021;9:630568. 10.3389/fbioe.2021.630568 33644022 PMC7902508

[22] Broman LM , Prahl Wittberg L , Westlund CJ , Gilbers M , Perry da Câmara L , Swol J , et al. Pressure and flow properties of cannulae for extracorporeal membrane oxygenation I: return (arterial) cannulae. Perfusion. 2019;34(Suppl 1):58–64. 10.1177/0267659119830521 30966910

[23] Schuler MJ , Becker D , Mueller M , Bautista Borrego L , Mancina L , Huwyler F , et al. Observations and findings during the development of a subnormothermic/normothermic long‐term ex vivo liver perfusion machine. Artif Organs. 2023;47(2):317–329. 10.1111/aor.14403 36106378

[24] Burke M , Pabbidi MR , Farley J , Roman RJ . Molecular mechanisms of renal blood flow autoregulation. Curr Vasc Pharmacol. 2014;12(6):845–858. 10.2174/15701611113116660149 24066938 PMC4416696

[25] Auvinen J , Tapio J , Karhunen V , Kettunen J , Serpi R , Dimova EY , et al. Systematic evaluation of the association between hemoglobin levels and metabolic profile implicates beneficial effects of hypoxia. Sci Adv. 2021;7(29):eabi4822. 10.1126/sciadv.abi4822 34261659 PMC8279517

[26] Hansell P , Welch WJ , Blantz RC , Palm F . Determinants of kidney oxygen consumption and their relationship to tissue oxygen tension in diabetes and hypertension. Clin Exp Pharmacol Physiol. 2013;40(2):123–137. 10.1111/1440-1681.12034 23181475 PMC3951849

[27] Hendriks KDW , Brüggenwirth IMA , Maassen H , Gerding A , Bakker B , Porte RJ , et al. Renal temperature reduction progressively favors mitochondrial ROS production over respiration in hypothermic kidney preservation. J Transl Med. 2019;17(1):265. 10.1186/S12967-019-2013-1 31409351 PMC6693148

[28] Meister FA , Czigany Z , Rietzler K , Miller H , Reichelt S , Liu WJ , et al. Decrease of renal resistance during hypothermic oxygenated machine perfusion is associated with early allograft function in extended criteria donation kidney transplantation. Sci Rep. 2020;10(1):17726. 10.1038/s41598-020-74839-7 33082420 PMC7575556

[29] Jenks CL , Zia A , Venkataraman R , Raman L . High hemoglobin is an independent risk factor for the development of hemolysis during pediatric extracorporeal life support. J Intensive Care Med. 2019;34(3):259–264. 10.1177/0885066617708992 28486865

[30] Moon YS , Ohtsubo S , Gomez MR , Moon JK , Nosé Y . Comparison of centrifugal and roller pump hemolysis rates at low flow. Artif Organs. 1996;20(6):579–581. 8817960

