# Supplementary material for: Impact of device variability and protocol differences on kidney function during normothermic machine perfusion: A comparative study using porcine and human kidneys
Source: Artif Organs. 2024 Aug 28;49(1):93–107. doi: 10.1111/aor.14851 (PMC11687212; doi:10.1111/aor.14851)
Supplement: Supplementary file 1 — Data S1.. [file AOR-49-93-s001.docx]

# Supplementary Methods

Pressure drop measurements for each cannula

Manometric pressure sensors (TruWave disposable pressure transducer (PX600F), Edwards Lifesciences Corp., Irvine, USA) were attached to the inlet and the outlet of each cannula. An adjustable pinch tube clamp was used as an artificial resistance to simulate a kidney’s resistance to perfusate flow (**Supplementary Figure 1**). Using a centrifugal pump (DP2, Medos Medizintechnik AG, Stolberg, Germany), a range of flows (0 – 1000 mL/min) was generated following a ramp profile with a red blood cell-based (hematocrit ≈ 0.25) perfusate at 37.0 °C (±0.5°C). The simultaneously measured pressures, as well as the perfusate flow measured by a factory-calibrated ultrasonic clamp-on flow sensor (ME 7PXL and TS410, Transonic Systems Inc., Ithaca, USA) were logged by means of a custom-designed data acquisition device and processed using MATLAB & Simulink (The MathWorks Inc., Natick, USA). After verification of their calibration and after subtraction of baseline measurements, the difference between pressure readings represents the pressure loss over the cannula. Pressure loss graphs were obtained by plotting the measured pressure loss versus the applied flow.

Detailed protocol for each group

**Kidney Assist protocol (KA)**

The Kidney Assist is a device designed for renal normothermic machine perfusion by XVIVO, Göteborg, Sweden. The disposable set contains a centrifugal pump, a membrane oxygenator, and a filter, all positioned in series in the circuit (**Figure 2A + B**). The oxygenator is also used as heat exchanger, connected to a water bath for temperature regulation. It is an open circuit, allowing for outflow of the perfusate from the renal vein into a reservoir underneath the kidney. The device is pressure-controlled, relying on feedback coming from a TruWave pressure sensor, and has a software-locked upper flow limit of 1000 mL/min. After reaching this flow limit, the device automatically lowers the set pressure to two-thirds of its original setting, continuing perfusion with the resultant flow. As per manufacturer instructions, infusions (8 µg/h Epoprostenol and 6 mL/h glucose 5%) were administered via the sample port.

**Modified Kidney Assist protocol (KA modified)**

A modified protocol was designed to overcome problems encountered during NMP of donor kidneys with a high vascular resistance. Such kidneys tend to have a very low initial flow rate, which does not allow their temperature to increase and, hence, will not trigger vasodilation to result in lower resistance and higher flow rates, thus maintaining a vicious circle of hypoperfusion and hypo-oxygenation. Introducing a shunt line from the oxygenator directly to the reservoir increases perfusate flow through the oxygenator, also used as a heat exchanger (**Figure 2A + B**). This way, the temperature control of the reservoir is improved, as well as the temperature regulation of the kidney, as it is partially submerged in the perfusate. Last, TruWave Pressure sensors are designed to have a counterflow in the pressure line. In patients, this keeps the pressure line clean of blood and thus prevents the formation of blood clots, which would result in inaccurate pressure measurements. Although no coagulation factors are added to our perfusate, the perfusate's red blood cells could still clog the pressure line during long-term perfusion. Therefore, a counterflow of 3 mL/h with 8 µg/h Epoprostenol was introduced to ensure an accurate pressure measurement. In order to precisely control the rate at which this counterflow would enter the perfusate, we did not use a pressurized infusion bag, but a syringe pump to drive it. An infusion of glucose 5% (6 mL/h) was administered via the oxygenator.

**PerLife protocol (PL)**

# The PerLife perfusion device is designed for hypothermic, subnormothermic, and normothermic machine perfusion of donor livers and kidneys. The PerKidney disposable set consists of two open circuits connected to the reservoir. The main circuit includes a roller pump and various sensors, and it is connected to the renal artery (Figure 2C + 3D). The device has a separate, parallel circuit for oxygenation, with two additional roller pumps, various sensors, an oxygenator, and a filter. Heating and cooling are managed through an isolated electronical heater/cooler plate located underneath the organ chamber with a temperature setpoint range of 4-37°C. The device is pressure-controlled by selecting a maximum (“systolic”) arterial pressure, with a software-defined flow limit of 500 mL/min. After reaching this flow limit, the machine continues perfusion with a perfusate flow of 500 mL/min with the resultant pressure. A counterflow of 3 mL/h with 8 µg/h Epoprostenol was introduced through the extra clinical-grade disposable TruWave pressure transducer. An infusion of glucose 5% (6 mL/h) was administered via infusion port, as per manufacturers instruction.

# Supplementary Figures

**Supplementary Figure 1**. Schematic overview of set-up for pressure drop measurements for the **A**. small (KP7-15FJ) and medium (KP10-20FJ) metal patch cannula recommended when using the Kidney Assist or PerLife perfusion device, and **B**. 8, 10, and 12 French straight cannulas recommended by the manufacturers when lacking an arterial patch.


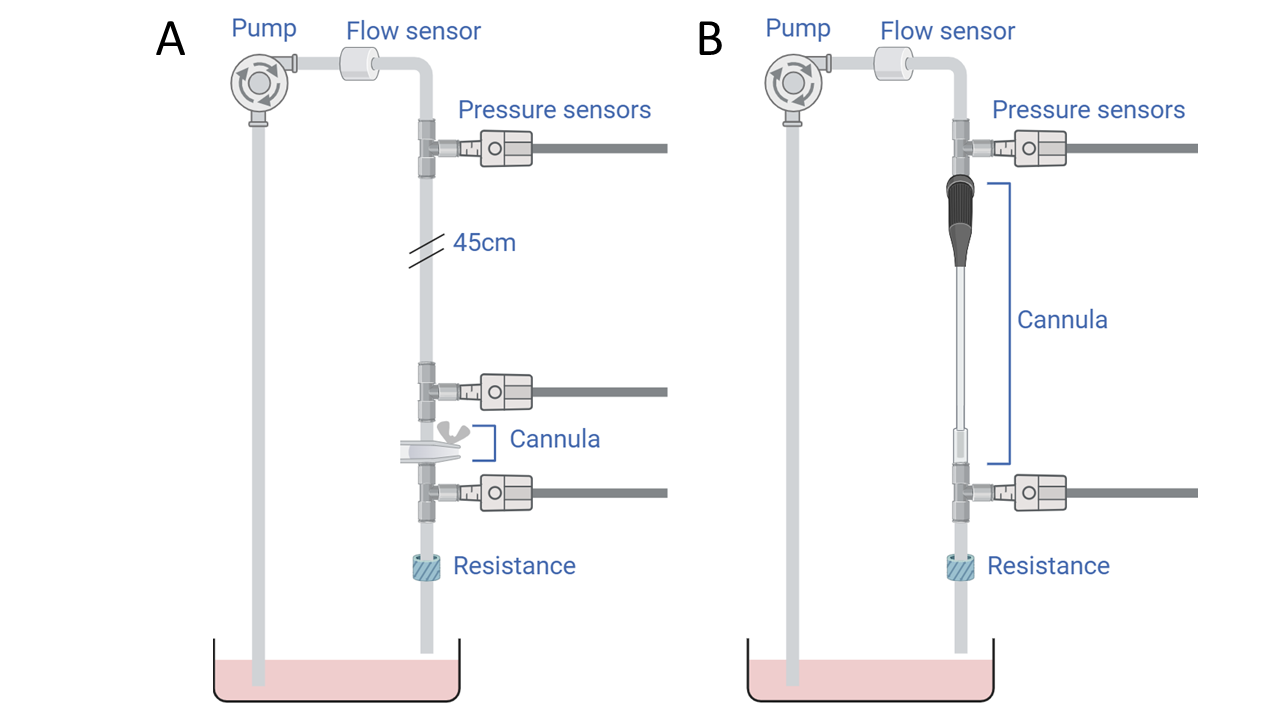


# Supplementary Tables

**Supplementary Table 1**. Perfusate composition. RBCs; red blood cells.

| **Component** | **Amount** | |
| --- | --- | --- |
| Autologous porcine RBCs | 336 | mL |
| NaCl solution 0.9% (Baxter, Utrecht, The Netherlands) | 505 | mL |
| Heparin 25.000IU/5mL (Leo Pharma, Ballerup, Denmark) | 0.5 | mL |
| Calcium gluconate 10% (B. Braun, Melsungen, Germany) | 8 | mL |
| Sodium bicarbonate 8.4% (B. Braun, Melsungen, Germany) | 40 | mL |
| Glucose 5% (Baxter, Utrecht, The Netherlands) | 28 | mL |
| Mannitol 15% (Baxter, Utrecht, The Netherlands) | 10 | mL |
| Magnesium sulfate 100 mg/mL (Eureco Pharma BV, Ridderkerk, The Netherlands) | 1 | mL |
| Aminoplasmal 10% (B. Braun, Melsungen, Germany) | 10 | mL |
| Sodium phosphate 3 mmol phosphate/mL (Pharmacy UMCG, Groningen, The Netherlands) | 0.3 | mL |
| Albuman (200 g/L human albumin) (CSL Behring, King of Prussia, USA) | 200 | mL |
| Sterile water (Baxter, Utrecht, The Netherlands) | 220 | mL |
| Cefazolin (Mylan, Canonsburg, USA) | 2 | g |
| Cernevit multivitamins (Baxter, Utrecht, The Netherlands) | 0.15 | g |
| Creatinin (Merck, Darmstadt, Germany) | 0.104 | g |
| **Infusions** | | |
| Epoprostenol (Flolan) (GSK, Amersfoort, The Netherlands) dissolved in NaCl solution 0.9% | 8 | µg/h |
| Glucose 5% | 6 | mL/h |

**Supplementary Table 2:** donor characteristics. Values shown are mean with standard deviation or percentage. HC; historical cohort, KA + WA; workaround protocol, WIT; warm ischemia time, CVA; cerebral vascular accident, CA; cardiac arrest, HMP-O2; oxygenated hypothermic machine perfusion, SCS; static cold storage.

| **Donor characteristics** | **HC 1** | **HC 2** | **HC 3** | **KA + WA 1** | **KA + WA 2** | **KA + WA 3** |
| --- | --- | --- | --- | --- | --- | --- |
| Donor # | 1 | 2 | 3 | 4 | 5 | 5 |
| Age (years) | 66 | 63 | 75 | 71 | 69 | 69 |
| Gender | Female | Male | Female | Male | Male | Male |
| BMI | 24 | 26 | 23 | 16 | 26 | 26 |
| Donor type | DCD | DCD | DCD | DCD | DCD | DCD |
| Kidney side | Right | Right | Right | Left | Right | Left |
| WIT (min) | 14 | 7 | 15 | 14 | 23 | 23 |
| Cold preservation time (min) | 1392 | 427 | 344 | 305 | 310 | 1105 |
| Cause of death | CVA | CA | CVA | CA | CA | CA |
| Preservation technique | HMP-O2 | SCS | SCS | SCS | SCS | HMP-O2 |
| **Reason for discard** |  |  |  |  |  |  |
| Poor kidney function in donor |  |  | V |  | V | V |
| Aneurysm renal artery | V |  |  |  |  |  |
| Logistical reasons |  | V |  |  |  |  |
| Macroscopic renal anomalies |  |  |  | V |  |  |

**Supplementary Table 3**. Equations that were applied to calculate renal function and viability.

| **Oxygen delivery (mlO_2_ * min^-1^  / 100g)**  $\frac{\left( Hb*{SO}_{2 arterial}*0.024794 \right)+\left( pO_{2arterial}*K \right)*Q}{g}*100$ |
| --- |
| **Oxygen consumption (mlO_2_ * min^-1^  /100g)**  $\frac{Hb*0.024794*\left( {SO}_{2 arterial}-{SO}_{2 venous} \right)+K*(pO_{2 arterial}-pO_{2 venous})*Q}{g}*100$ |
| **Fractional sodium excretion (%)**   |
| **Metabolic coupling (mmol Na * mmol O2^-1^/100g)**  $\left( \frac{\frac{\left( \left( \frac{U_{Cr}*U}{P_{Cr}} \right)*P_{Na}*0,001 \right)-\left( U_{Na}*U*0,001 \right)}{(Hb*0.024794*\left( {SO}_{2 arterial}-{SO}_{2 venous} \right)+K*(pO_{2 arterial}-pO_{2 venous})*Q)*0,04464}}{g} \right)*100$ |
| g: kidney weight (gram).  Hb: hemoglobin content (mmol/L).  K: solubility constant of oxygen in H_2_O at 37°C (0.0225 mlO_2_ per kPa).  P_Cr_: perfusate creatinine concentration (mmol/L).  P_Na_: perfusate sodium concentration (mmol/L).  pO_2_: partial oxygen pressure (kPa).  Q: renal blood flow (dL/min).  SO_2_: hemoglobin saturation (%).  U: urine production rate (mL/min).  U_Cr_: urine creatinine concentration (mmol/L).  U_Na_: urine sodium concentration (mmol/L). |
